# Supplementary material for: Automatic Detection of the Circulating Cell-Free Methylated DNA Pattern of GCM2, ITPRIPL1 and CCDC181 for Detection of Early Breast Cancer and Surgical Treatment Response
Source: Cancers (Basel). 2021 Mar 18;13(6):1375. doi: 10.3390/cancers13061375 (PMC8002961; doi:10.3390/cancers13061375)
Supplement: Supplementary file 1 [file cancers-13-01375-s001.pdf]

Supplementary Files

Automatic Detection of the Circulating Cell-Free Methylated DNA Pattern of *GCM2*, *ITPRIPL1* and *CCDC181* for Detection of Early Breast Cancer and Surgical Treatment Response

Sheng-Chao Wang, Li-Min Liao, Muhamad Ansar, Shih-Yun Lin, Wei-Wen Hsu and Chih-Ming Su, Yu-Mei Chung, Cai-Cing Liu, Chin-Sheng Hung and Ruo-Kai Lin

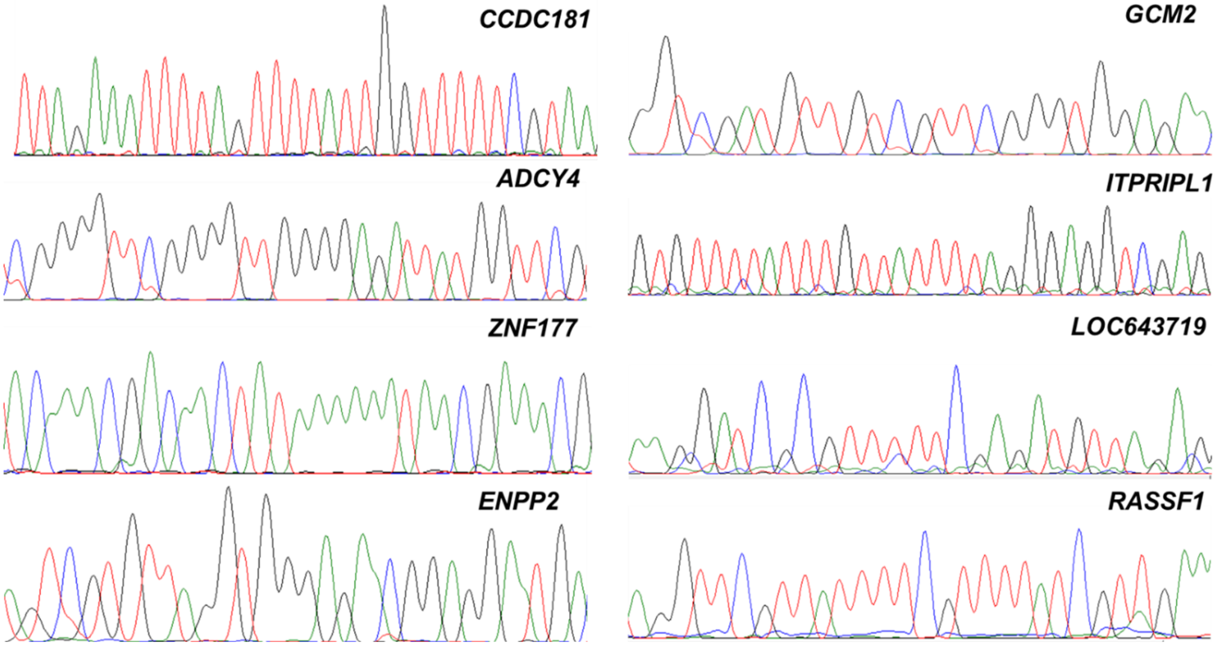

Figure S1. Representative standard sequencing diagram for bisulfite direct sequencing of the *CCDC181, GCM2, ITPRIPL1, ENPP2, LOC643719, ZNF177, ADCY4* and *RASSF1* genes.

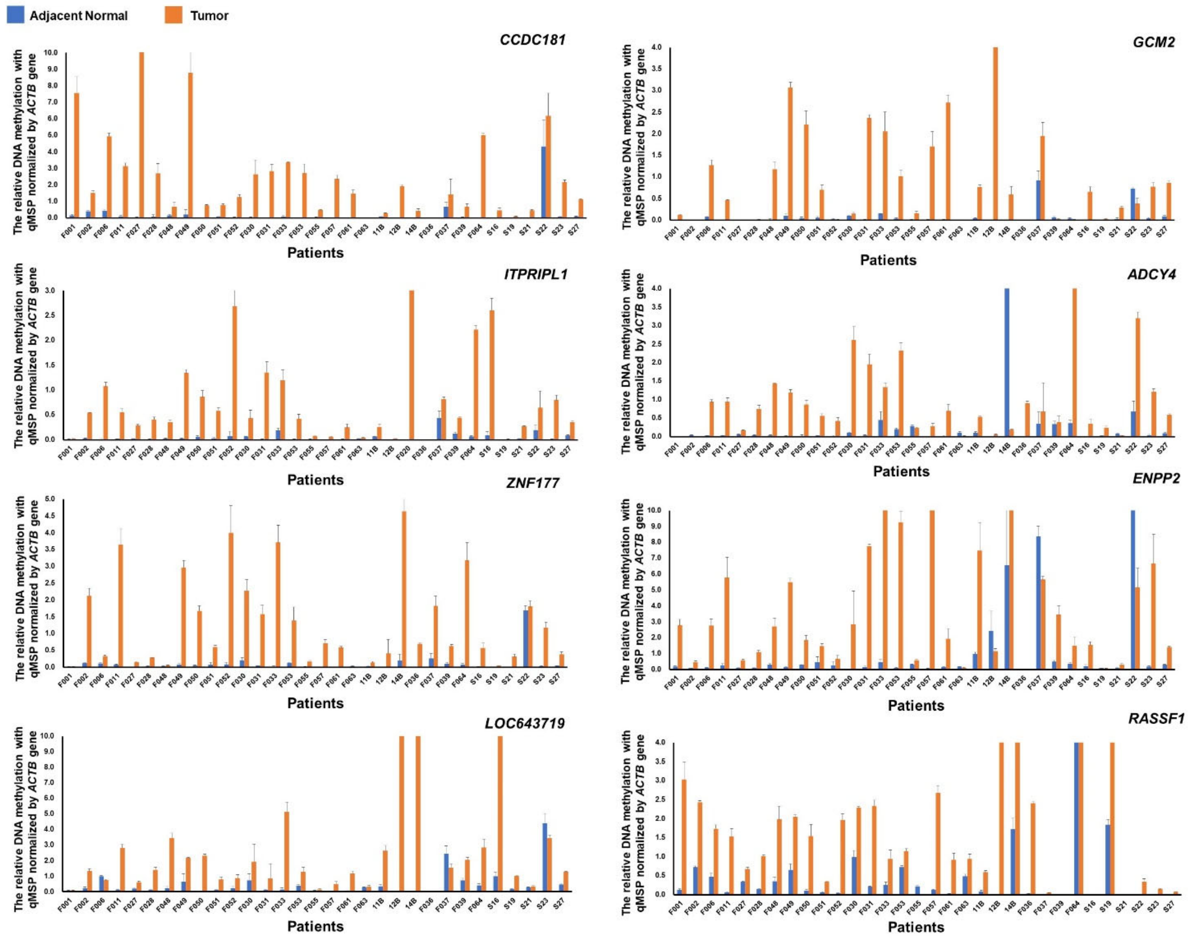

Figure S2. Representative figures showing the DNA methylation levels of the candidate genes *CCDC181, GCM2, ITPRIPL1, ENPP2, LOC643719*, *ZNF177*, *ADCY4* and *RASSF1* using qMSP in breast cancer patients.

Table S1. The clinical parameters of breast cancer patients for plasma ccfDNA analysis.

Characteristics	Manual 0.2 mL Plasma	Manual 0.5 mL Plasma	Automatic Labturbo 1.6 mL Plasma	Automatic Duo Prime 1.6 mL Plasma
	N (%)	*N* (%)	*N* (%)	*N* (%)
Overall	45	34	63	57
Type				
≤45	19(42.2)	13(38.2)	9(14.3)	10(17.5)
>45	26(57.8)	21(61.8)	54(85.7)	47(82.5)
Type				
DCIS	3(6.7)	10(29.4)	7(11.1)	1(1.8)
IDC	40(88.9)	22(64.7)	47(74.6)	48(84.2)
ILC	1(2.2)	2(5.9)	1(1.6)	2(3.5)
Others	1(2.2)	0	8(12.7)	6(10.5)
Tumor Stage				
0, I and II	32(71.1)	29(85.3)	52(82.5)	44(77.2)
III and IV	13(28.9)	5(14.7)	11(17.5)	13(22.8)
Tumor Size				
T0–T2	38(84.4)	31(91.2)	56(88.9)	49(86.0)
T3–T4	7(15.6)	3(8.8)	7(11.1)	8(14.0)
Lymph node				
N = 0	16(35.6)	22(64.7)	31(49.2)	30(52.6)
N > 0	29(64.4)	1235.3)	32(50.8)	27(47.4)
ER				
Negative	21(46.7)	7(20.6)	19(30.2)	19(33.3)
Positive	24(53.3)	27(79.4)	44(69.8)	38(66.7)
PR				
Negative	20(44.4)	11(32.4)	21(33.3)	22(38.6)
Positive	25(55.6)	23(67.6)	42(66.7)	35(61.4)
HER2				
Negative	35(77.8)	26(76.5)	46(73.0)	35(61.4)
Positive	10(22.2)	8(23.5)	17(27.0)	22(38.6)
Ki-67				
High	24(53.3)	24(70.6)	39(61.9)	39(68.4)
Low	21(46.7)	10(29.4)	24(38.1)	12(21.1)
n.d.	-	-	-	6(10.5)

Table S2. List of primer sequences and conditions used in the present study.

Gene	Primer	5′→3′Sequences	Application	Size (bp)	Tm (℃)
BACTIN	Forward<br>Reverse<br>Probe	TGGTGATGGAGGAGGTTTAGTAAGT<br>AACCAATAAAACCTACTCCTCCCTTAA<br>ACCACCACCCAACACACAATAACAAACACA	Input DNA control (No CpG)	132	60
CCDC181	Forward<br>Reverse<br>Probe	TTTTATTGGTTTTTCGTAAGTATCG<br>CATAACAACAACGTACCTCTACGTC<br>TCGGGAGGGGTCGGTGGTTTGAG	MSP-M	143	60
GCM2	Forward<br>Reverse<br>Probe	GAGATAGGGCGGAGTTTTTC<br>CTTAACCGCGATACTAAACGTT<br>TCCACCCGAACGACAACATCGACC	MSP-M	105	60
ITPRIPL1	Forward<br>Reverse<br>Probe	GAGTGTAGTTGATAGTAGGTACGGC<br>GTAAATTTACTAAAAAAATAAAAAAACCGT<br>CACACTCTCCGCTACTCGACCTCCCTA	MSP-M	106	60
ZNF177	Forward<br>Reverse<br>Probe	TTTAGTTGTTGGTCGGAAGC<br>CGACCTCACTAATAAAACGCA<br>AACGAAACGACGAACGCCCACTTC	MSP-M	128	60
LOC643719	Forward<br>Reverse<br>Probe	CCTCTAACATCTCGAAAAACG<br>TGGAGTGTTATAAATTTTATTATCGT<br>TC GGG TTC GT TTT TAG GAT ACG GAG TT	MSP-M	116	60
ENPP2	Forward<br>Reverse<br>Probe	TAAAAGGTTTTTTAAGAATTTTCGA<br>TAAAAATCAAACATATCCCCCG<br>TCCCACCTAACACGACTAAAACGAACT	MSP-M	101	60
ADCY4	Forward<br>Reverse<br>Probe	GAGAAAAGTTTAGGTGGGGTTC<br>TAAATCTCGTAAAAAAAATCTTCGC<br>CCCCAACCCCGAACCCCCGAAA	MSP-M	148	60
RASSF1	Forward<br>Reverse<br>Probe	GGTAGTTAAGGGGTAGCGTAGTC<br>TTCAACGATAAAACGAAAATAACG<br>CCCCTCTACCGCGACTTAACCCGC	MSP-M	105	60

Table S3. Gene list of the 160 hypermethylated genes that were commonly found in Taiwanese and TCGA cohorts.

Status	Genes
Aberrant DNA methylated genes in cancers reported previously	*AHRR, BARHL2, BOLL, C12orf68, C14orf23, C17orf64, C9orf122, CCDC36, CCDC8, CLIP4, CPXM1, CRHR2, CRYGD, CSDAP1, DPP6, GNG4, HLA-L, HOXA4, HOXD8, ILDR2, KCNK9, MIR129-2, MMP9, MYO15B, NES, NPTX2, NRXN1, NT5E, OLIG3, OTX2OS1, PGLYRP2, PHOX2A, PRDM14, RCN3, SCG5, SCRT2, SEMA6C, SKI, SLITRK1, SOX2OT, SPTBN4, TBR1, TIMP2, TTBK1, TTC28, TULP1, TXNRD1, VWC2, ZNF454, ZNF572*
Breast cancer-associated genes reported previously	*CPEB1, CYTL1, DOCK2, ESRRG, FLI1, FRZB, GRASP, GRIA1, HOXD9, LHX1, MAML3, MEIS2, NCALD, NKX2-1, NPAS4, OCA2, PDX1, RGS17, RGS20, SALL1, SSTR4, TMEM97, TNFAIP8L3, TRABD, VGLL4, WNT3A*
Novel aberrant DNA methylated genes	*ADCY4, ALX1, BCAT1, C12orf42, C1orf114 (CCDC181), CFTR, CHST11, CHST3, CLDN9, CLEC14A, COL11A2, CRYM, CSMD3, DBX1, DMRTA2, DNM3, DPF1, EBF1, EMX1, ENPP2, EPHX3, EVX2, F2RL3, FAM38B, FOXD3, FSD1, GALR1, GCK, GCM2, GJD2, GRIN1, H2AFY, HCK, HNF1B, HPCAL4, HTR6, IRX1, ITGA5, ITPRIPL1, KCNC3, LHX4, LHX8, LOC643719, LOC646999, MIR663, NID2, NR5A2, NRXN2, NXPH1, OTX1, OTX2, PITX2, POU3F3, POU4F2, PRDM13, PRKAR1B, PRKCB, PRKCE, PRRT1, PTPRN, RNF220, SEZ6L2, SIM1, SLC23A2, SNAP25, SOSTDC1, SRGAP3, SRRM3, SSPO, TAC1, TFAP2B, TLX1, TMEM145, TRH, TRIM46, TRIM71, TRIP10, VANGL2, WIT1, ZIC5, ZNF177, ZNF662, ZSCAN18, TBXT*

Table S4. The functions of the candidate hypermethylated genes.

Gene	Functions
C1orf114 (CCDC181)	*CCDC18* is a microtubule-binding protein and a structural component of cilia and sperm flagella [78].
GCM2	*GCM2* is a gene encoding a transcription factor required for parathyroid development. The C-terminal conserved inhibitory domain mutation of GCM2 can cause primary hyperparathyroidism [80].
ITPRIPL1	Unknown
ZNF177	Unknown
ADCY4	*ADCY4* is a member of the family of adenylate cyclases, mediating cyclic adenosine monophosphate (cAMP) synthesis inhibition of caspase-11 inflammasome activation in macrophages [81]
ENPP2	Autotaxin (*ATX, ENPP2*) is a secreted glycoprotein that catalyzes the extracellular production of lysophosphatidic acid (LPA), a growth-factor-like phospholipid that is further regulated by phospholipid phosphatases (PLPP) [79].
LOC643719 (SCGB1B2P)	Pseudogene

Table S5. The methylation level of CCDC181, GCM2, ITPRIPL1 in different types of cancer patients

**Taiwan**	***CCDC181***			***GCM2***			***ITPRIPL1***		
Cancer Type	Avg T	Avg N	Pair [1] T/N > 10	Avg T	Avg N	Pair T/N > 10	Avg T	Avg N	Pair T/N > 10
Breast *N* = 109	1.27	0.09	68.0%	0.44	0.18	60.0%	0.55	0.07	68.0%
Colorectal *N* = 24	0.62	0.10	20.8%	0.33	0.07	12.5%	0.08	<0.01	37.5%
Esophageal *N* = 16	2.98	0.10	56.3%	0.67	0.02	25.0%	0.54	<0.01	50.0%
Lung *N* = 33	0.31	0.06	12.1%	0.08	0.01	33.3%	0.01	<0.01	60.6%
Endometrial *N* = 15	2.79	0.13	66.7%	0.40	0.22	20.0%	0.37	0.01	20.0%
TCGA	*CCDC181*			*GCM2*			*ITPRIPL1*		
Cancer Type	Avg β(T)	Avg β(N)	Avg β (T−N)	Avg β(T)	Avg β(N)	Avg β (T−N)	Avg β(T)	Avg β(N)	Avg β (T−N)
Breast	0.51	0.08	0.44	0.43	0.08	0.35	0.45	0.12	0.33
Colon	0.58	0.19	0.39	0.50	0.13	0.37	0.23	0.08	0.15
ESCC	0.37	0.13	0.24	0.46	0.17	0.29	0.21	0.11	0.10
Stomach	0.35	0.17	0.19	0.37	0.28	0.09	0.23	0.08	0.15
Liver	0.26	0.08	0.19	0.20	0.09	0.11	0.37	0.21	0.16
Lung AD	0.31	0.09	0.22	0.37	0.14	0.24	0.30	0.18	0.12
Lung SQ	0.42	0.05	0.37	0.28	0.08	0.19	0.33	0.13	0.20
Pancreas	0.13	0.04	0.09	0.18	0.08	0.11	0.19	0.14	0.06
Uterine	0.72	0.06	0.66	0.25	0.08	0.17	0.15	0.15	0.00
Ovarian	0.16	-	-	0.10	-	-	0.11	-	-

[1] The results of the paired T/N ratio were calculated from qMSP analysis in tumors (T) in comparison to adjacent normal tissues (N) of cancer patients.

Table S6. The methylation of candidate genes in relation to the clinical parameters in The Cancer Genome Atlas (TCGA) breast cancer patients[1].

**Characteristics**		***CCDC181***			***GCM2***			***ITPRIPL1***		
**Methylation**	***N***	**High (%)**	**Low (%)**	***p***	**High (%)**	**Low (%)**	***p***	**High (%)**	**Low (%)**	***p***
Age	547	52.3	47.7	0.041	50.1	49.9	0.052	50.6	49.4	0.759
>45	116	41.4	58.6		39.7	60.3		52.6	47.4	
<45	656	50.2	49.8	0.451	48.3	51.7	1.000	50.9	49.1	1.000
Gender	7	71.4	28.6		42.9	57.1		57.1	42.9	
Female	507	49.9	50.1		47.3	52.7		49.3	50.7	
Male	32	59.4	40.6	0.563	68.8	31.3	0.063	68.8	31.3	0.094
Race	110	49.1	50.9		48.2	51.8		52.7	47.3	
White	153	47.1	52.9	0.221	40.5	59.5	0.011	45.8	54.2	0.074
Asian	426	53.1	46.9		52.6	47.4		54.2	45.8	
Black or African American	372	54.0	46.0	0.035	50.5	49.5	0.210	53.5	46.5	0.159
Menopause state	291	45.7	54.3		45.4	54.6		47.8	52.2	
Premenopause	9	66.7	33.3	0.505	66.7	33.3	0.326	66.7	33.3	0.506
Postmenopause	654	50.2	49.8		48.0	52.0		50.8	49.2	
Lymph node	479	47.8	52.2	0.042	46.1	53.9	0.111	50.3	49.7	0.659
N > 1	174	56.9	43.1		53.4	46.6		52.3	47.7	
N = 0	479	56.8	43.2	<0.001	52.6	47.4	<0.001	51.8	48.2	0.705
Distant metastasis	145	29.7	70.3		35.2	64.8		49.7	50.3	
Yes	423	57.9	42.1	<0.001	51.1	48.9	0.085	52.2	47.8	0.606
No	198	34.8	65.2		43.4	56.6		50.0	50.0	
Stage	97	59.8	40.2	0.048	64.9	35.1	<0.001	64.9	35.1	0.003
I and II	566	48.8	51.2		45.4	54.6		48.6	51.4	
III and IV	455	51.6	48.4		49.9	50.1		55.8	44.2	
ER	133	50.4	49.6		47.4	52.6		38.3	61.7	
Positive	23	60.9	39.1		47.8	52.2		52.2	47.8	
Negative	13	30.8	69.2		38.5	61.5		30.8	69.2	
PR										
Positive										
Negative										
HER2				0.160			0.491			0.003
High										
Low	39	35.9	64.1		35.9	64.1		43.6	56.4	
Histological Type										
IDC										
ILC										
Mix Histology										
Mucinous										
Others										
